# Identification of Key Residues for pH Dependent Activation of Violaxanthin De-Epoxidase from *Arabidopsis thaliana*

**DOI:** 10.1371/journal.pone.0035669

**Published:** 2012-04-27

**Authors:** Christian Fufezan, Diana Simionato, Tomas Morosinotto

**Affiliations:** 1 Institute for Biology and Biotechnology of Plants, University of Muenster, Münster, Germany; 2 Biology Department, University of Padova, Padova, Italy; University of California Davis, United States of America

## Abstract

Plants are often exposed to saturating light conditions, which can lead to oxidative stress. The carotenoid zeaxanthin, synthesized from violaxanthin by Violaxanthin De-Epoxidase (VDE) plays a major role in the protection from excess illumination. VDE activation is triggered by a pH reduction in the thylakoids lumen occurring under saturating light. In this work the mechanism of the VDE activation was investigated on a molecular level using multi conformer continuum electrostatic calculations, site directed mutagenesis and molecular dynamics. The pK_a_ values of residues of the inactive VDE were determined to identify target residues that could be implicated in the activation. Five such target residues were investigated closer by site directed mutagenesis, whereas variants in four residues (D98, D117, H168 and D206) caused a reduction in enzymatic activity indicating a role in the activation of VDE while D86 mutants did not show any alteration. The analysis of the VDE sequence showed that the four putative activation residues are all conserved in plants but not in diatoms, explaining why VDE in these algae is already activated at higher pH. Molecular dynamics showed that the VDE structure was coherent at pH 7 with a low amount of water penetrating the hydrophobic barrel. Simulations carried out with the candidate residues locked into their protonated state showed instead an increased amount of water penetrating the barrel and the rupture of the H121–Y214 hydrogen bond at the end of the barrel, which is essential for VDE activation. These results suggest that VDE activation relies on a robust and redundant network, in which the four residues identified in this study play a major role.

## Introduction

Nearly all life on earth depends directly or indirectly on light as a source of energy. Photosynthetic organisms need to harvest light with the highest possible efficiency to optimize growth, yet, when exposed to intense illumination such an efficient harvesting increases the probability of radiation damage due to over-excitation and the consequent generation of reactive oxygen species [1]. Plants and algae are exposed to a variable environment and therefore need to modulate light harvesting efficiency to avoid high light radiation damages. A central role in this process is played by the xanthophyll cycle, i.e. in the conversion of the di-epoxide xanthophyll violaxanthin into the epoxide-free zeaxanthin, catalyzed by the enzyme Violaxanthin De-Epoxidase (VDE) [2]. The mono-epoxide anteraxanthin is the intermediate step of the reaction which is normally not accumulated *in vivo*. Zeaxanthin enhances the photoprotection capacity and it has been shown to increase the quenching of excited Chlorophyll states (both singlet and triplet) as well as the scavenging of reactive oxygen species eventually formed [1], [3]. Consistent with the role of zeaxanthin in photoprotection, mutants depleted of VDE showed increased susceptibility to high light and a reduced fitness in natural conditions [4]. In low light conditions zeaxanthin is converted back to violaxanthin by the stromal enzyme Zeaxanthin Epoxidase (ZE) [5]. The xanthophyll cycle is a mechanism found in many different photosynthetic eukaryotes, such as plants, green algae and diatoms [6].

In order to maximize photosynthetic efficiency in all environmental conditions it is however essential that an energy dissipation pathway is activated only when needed, otherwise growth would be reduced in light limiting conditions. When light energy is absorbed in excess and saturates the capacity for energy conversion the pH of the lumen drops. The acidification of this compartment is a key feed-back signal which triggers two fundamental responses to excessive illumination, the activation of the Non Photochemical Quenching (NPQ) through PsbS protonation [7] and the induction of the zeaxanthin synthesis via the activation of the Violaxanthin De-Epoxidase (VDE)[8]. VDE activation is associated with a conformational change [9] and the protein association to the thylakoids membrane, where its substrate violaxanthin is found [10], [11].

VDE belongs to a multigenic protein family called lipocalins, whose members are characterized by a conserved structural organization with an 8 strands β-barrel and often bind hydrophobic molecules [12]. VDE also has two additional domains with no clear homology to any other known protein. These were named after their peculiar amino acid composition as Cysteine-rich and Glutamate-rich domains [12], [13]. The VDE lipocalin domain (VDE_cd_) structure has been recently resolved from crystals grown at acidic and neutral pH [14] and these structures illustrate the pH dependent conformational changes associated with the protein activation. Based on the analysis of pH 5 structure it was also suggested that active VDE is a dimer, where both violaxanthin rings can dock and react at once [14], [15]. In each monomer a binding site for ascorbate, the second substrate of the reaction providing the reducing power for de-epoxidation reaction, was also identified [15].

In this work the molecular mechanism for the pH dependent activation of VDE was investigated. The pK_a_ values of residues in the VDE_pH7_ structure were calculated using multi conformer continuum electrostatics [16]–[18] to identify potential targets to elucidate the residues responsible for the VDE molecular activation switch. Five candidate residues having a pK_a_ between 5.2 and 7 were further investigated. Site directed mutagenesis was used to create VDE variants that are not able to change ionization state of these five residues, showing in four cases (D98, D117, D206, H168) a significant reduction in protein activity whereas variants of D86 did not show any alteration. Sequence analysis of VDEs further supports the identification of the activation candidates in the light of the differences in pH activation range between plants and diatoms. Molecular dynamics was also used to investigate the integrity of the VDE_pH7_ structure and how the structure changes in the very beginning of the activation. The protonation of the same four residues leads to the destabilization of the protein structure whereas the protonation of D86 did not. All data shown consistently suggest the identification of these residues as major players in first steps of VDE pH dependent activation.

## Results

**Figure 1:**
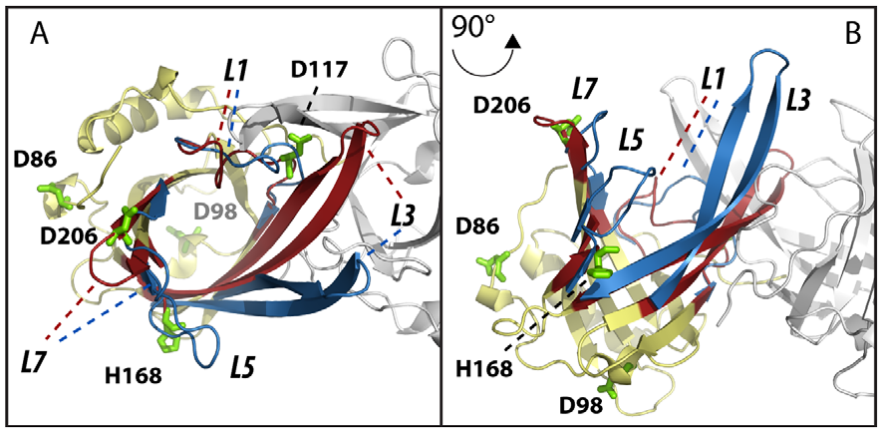
Candidate residues responsible for pH dependent VDE conformational change. Superimposition of the VDE central domain structure (VDE_cd_) at pH 7 and 5. The regions where structure organization is conserved in the two structures are shown in yellow while the ones showing differences are highlighted in red and blue for the inactive (VDE_pH7_) and active (VDE_pH5_) structure, respectively. The second monomer of the active dimeric structure is shown in grey to visualize the dimerization interface. Major rearrangements upon activation involve loops L1, L3, L5, L7. Putative residues involved in the pH dependent conformational change (D86, D98, D117, D206, H168) are shown as green sticks. A) Top view of the monomers. B) same as A after x axis rotation of 90°.

Violaxanthin De-Epoxidase (VDE) activation is pH dependent and known to require a conformational change [9]. The recently solved molecular structure of the VDE lipocalin domain from crystals grown at pH 5 and 7 showed several details of this conformational change ([14], Figure 1A, 1B). The major rearrangements of the tightly packed barrel structure of VDE_pH7_ occur on one side of the protein and involve the loops L1, L3, L5 and L7 [14]. These modifications result in a major change in the accessibility of the lipocalin cavity, which is accessible to the substrate violaxanthin only in the active form at low pH [14], [15] (Figure S1). This conformational change is likely also a fundamental pre-requisite for protein dimerization at pH 5. In fact, most protein-protein contacts in the dimer ([14] and Figure S2) are not possible in the VDE_pH7_ structure. For example, at pH 7 D114 in loop L1 is hydrogen-bonded to Y198 in the barrel cavity. Following the loop rearrangement at pH 5, D114 is involved instead in a salt bridge with R138 and hydrogen-bonded to D114 of the adjoining monomer with this interaction likely to be fundamental for the dimer stabilization (see below and [14]).

### Candidates for the VDE Activation on a Molecular Level

In this study the pK_a_ values of residues of the inactive VDE were determined using Multi Conformer Continuum Electrostatic (MCCE2 [16]) calculations (Table 1, Table 1) to identify residues that might be implicated in the molecular activation of VDE. Five residues with pK_a_ values between 5.0 and 7.0 were identified: D86 (pK_a_: 5.0), D98 (pK_a_: 5.3), D117 (pK_a_: 5.3), D206 (pK_a_: 5.2) and H168 (pK_a_: 7.0). These five candidate residues change their protonation state by 50 or more percent when pH is decreased from 7 to pH 5.2, the optimal pH for VDE activity [19]. The residues are distributed over the protein structure and are not concentrated in any particular region of the protein. Only D117 is found close to the monomer-monomer interface in the dimeric protein at pH 5 (Figure 1, Figure S2). All five residues are surface exposed and their pK_a_ values are close to the reference values determined in water. The same type of continuum electrostatic calculations was performed on the VDE structure at pH 5 (Table S2). Since the activated dimer is associated to the membrane, the *in vivo* pK_a_ values will certainly change for residues that are close to the membrane interface. However, those calculated values still provide useful information since they reflect the changes induced by the structural rearrangement and dimerization.

**Table 1:** pK_a_ of selected VDE residues.

Residue	pK_a_	std
ASP86	5.0	0.1
ASP98	5.3	0.2
ASP117	5.3	0.3
HIS168	7.0	0.3
ASP206	5.2	0.1

pK_a_ values of all ionizable residues are shown in Table S1. Here are reported the ones identified as putatively responsible of the pH dependent activation, having pK_a_ between 5 and 7 in VDE_pH7_.

### Experimental Support by Site Directed Mutagenesis

Evidently, pK_a_ calculations come with an error that has to be taken into account [16]. For this reason, the potential candidates were further investigated by replacing them with non-protonable groups using site directed mutagenesis. These VDE variants (D86A, D98L, D117A, H168A and D206I) will not change their protonation state when the protein is incubated at pH 5.2. The activity of all mutants relative to the WT was evaluated by measuring their ability to convert violaxanthin to zeaxanthin. The results in Table 2 and Figure S3 show that all but one mutant exhibit a significant reduction in enzymatic activity. The biggest impact was observed with the exchange of H168, while no effects could be measured in the variant D86A. All mutations showing a significant effect where combined afterwards and protein activity decreased even further, reaching 6% of WT activity. However, significant enzymatic activity was still detectable, suggesting that although these residues are important for VDE activity the protein is not completely inactive in their absence.

**Table 2:** Enzyme activity of VDE variants compared to WT.

ACTIVITY	% WT ± SD	ACTIVITY	% WT ± SD
**D86A**	81 ± 18	**D206I**	44 ± 8
**D98L**	59 ± 5	**D98L/D117A**	41 ± 10
**D117A**	40 ± 14	**D98L/D117A/D206I**	16 ± 10
**H168A**	20 ± 8	**D98L/D117A/D206I/H168A**	6 ± 3

The enzyme activity of all generated mutants is reported, as determined with a spectroscopic method exploiting the different absorption of violaxanthin and zeaxanthin at 502 nm [24]. Activity is expressed as % of WT control sample, together with SD (n = 5). The protein amount employed for enzymatic essays was verified to be equivalent for WT and mutants by Western blotting using specific antibodies.

### Electrostatic Interaction Node Maps

**Figure 2:**
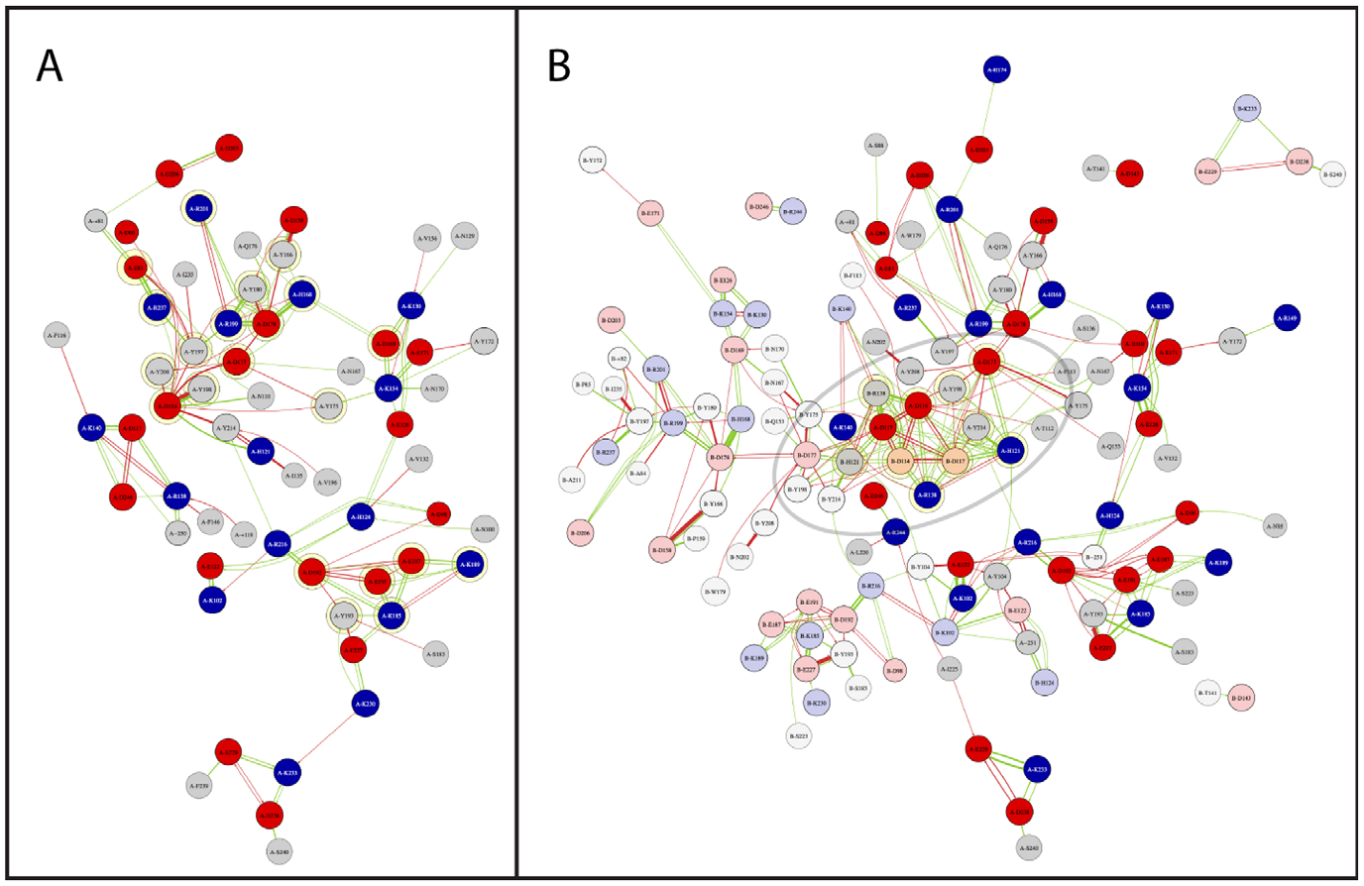
Electrostatic interaction node maps and position of H121-Y214 hydrogen bond. Electrostatic interaction node maps generated from the results of the multi conformer continuum electrostatic calculations for A) the VDE_pH7_ and B) the VDE_pH5_. Acids and bases are shown as red and blue, respectively. The width of the edge is scaled according to the strength of the interaction. Green and red edges indicate stabilizing or de-stabilizing interactions, respectively. Electrostatic interaction node maps of the VDE_pH5_ show residues of chain B opaque. A contact map of the two subunits is shown in Figure S2. Interaction are only shown if the absolute electrostatic interaction is greater than 0.4 kcal mol^−1^. The graph densities are 0.059 and 0.029, for VDE_pH7_ (A) and VDE_pH5_ (B) node maps, respectively. Interaction cutoff is 0.2 kcal mol^−1^. Major interaction hubs, representing *k-cores* of 4 or 7 and 6 in the VDE_pH7_ (A) or VDE_pH5_ (B) form, respectively, are highlighted with yellow circles.

**Figure 3:**
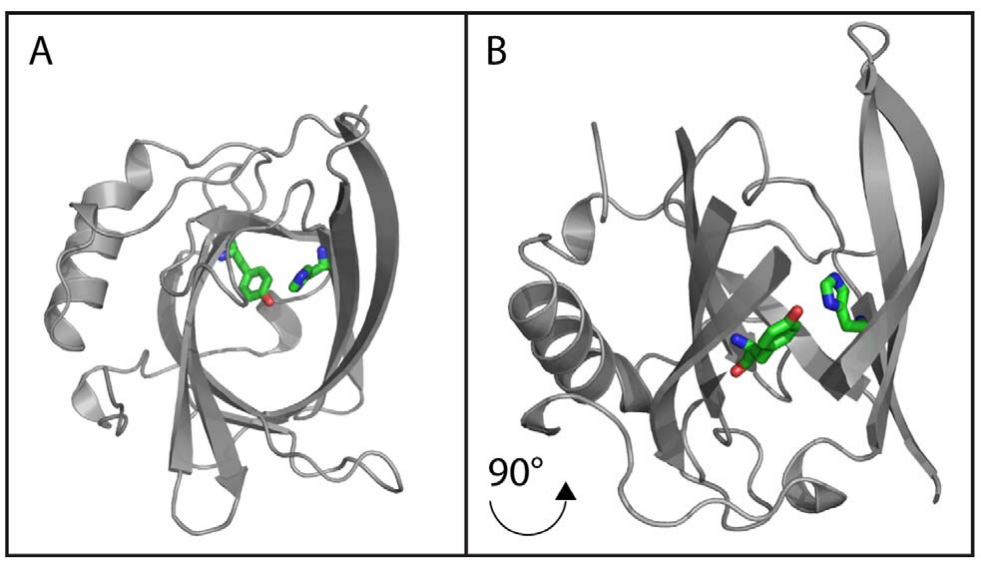
Position of H121-Y214 hydrogen bond in VDE_pH7_ structure. A) Top view of the inactive monomer, shown as grey cartoons. H121 and Y214 are shown as red sticks. B) Same as C after x axis rotation of 90° rotation. A part of the β-barrel is not shown for the sake of clear visualization of the H121–Y214 hydrogen bond.

Electrostatic interaction node maps can be used to visualize the origin of unusual pK_a_ values and interaction hot spots. Figure 2 shows the averaged electrostatic interactions in the VDE structure at pH 7 and pH 5 (Figure 2A, 2B). A *k*-cores decomposition (http://arxiv.org/abs/cs.DS/0310049) can be exploited to identify the most relevant residues/nodes in the map (Table S3). The VDE_pH7_ has two major cohesive subgroups with a *k-core* value of 4: *A*) E81, R237, R201, Y208, Y197, R199, Y180, Y166, D114, Y198, D177, D178, D169, Y175 and H168 and *B*) D192, E187, E191, Y193, K189 and K185 (upper and lower part of Figure 2A). Figure 2B shows the same type of electrostatic interaction node map for the VDE_pH5_. Positions of residues are kept fixed between the two node map figures. The VDE_pH5_ node map shows only one major subgroup with a *k-core* value of 7 and in the case of B-H121 & B-R138 of 6. The higher *k-core* values are due to the dimerization process, which brings the subgroup *A* from each monomer into close contact to each other. In fact this electrostatic interaction hub represents the major dimer stabilizing electrostatic interaction. In this hub, the very close proximity of residues D114 and D117 from each monomer gives rise to several strong unfavorable electrostatic interactions, shown as red arrows. These accumulations of negative charges are compensated by R138 and H121 from each monomer. The strongest compensation is thereby provided from the bases of the other monomer. Comparing node maps VDE_pH7_ and VDE_pH5_, it is eminent that while R138 is already stabilizing D117 in VDE_pH7_ (Figure 2A) and thus is already suited to take its role in the acidic form, H121 is not. In VDE_pH7_ H121 shows a pK_a_ of 4.2 and is therefore at pH 7 neutral, not offering any substantial compensating positive charge. This unusual low pK_a_ of H121 in VDE_pH7_ is due to a strong hydrogen bond with Y214, which will have to be eliminated to expose H121 to the solvent, allowing its protonation and insertion in the major stabilizing hub in the acidic form. This analysis thus underlines that VDE activation requires a reorganization of electrostatic interactions, which necessarily requires the preliminary disruption of the strong hydrogen bond between H121–Y214. This hydrogen bond is located at the end of the closed hydrophobic core of VDE_pH7_ (Figure 3) and it can be expected that a loosening of the tight hydrophobic barrel structure has to occur prior to dimerization. A loosening will allow water to enter the barrel and thus weaken the hydrogen bond itself.

### Stability of Inactive VDE during Molecular Dynamics

The VDE_pH7_ shows a beta sheet barrel as central motif, holding at its end H121 and Y214 (Figure 3). From the VDE_pH5_ structure and the electrostatic interaction node maps it seems clear that the H121–Y214 hydrogen bond needs to be disrupted to allow the formation of the major electrostatic interaction hub described above. This breakage is also required for the rearrangement of loops L1 and L3, which is a necessary pre-requisite for protein dimerization [14]. Considering H121 key relevance in the major electrostatic interaction hub and its role in the activation, the H121–Y214 hydrogen bond disruption was monitored during molecular dynamics simulations to estimate the structural integrity of VDE_pH7_. The beginning of the protein conformational change can also be evidenced by the increased amount of water molecules within the hydrophobic core structure. For this reason the number of solvent contacts within 0.5 nm of the Y214 hydroxy hydrogen was also evaluated.

**Figure 4:**
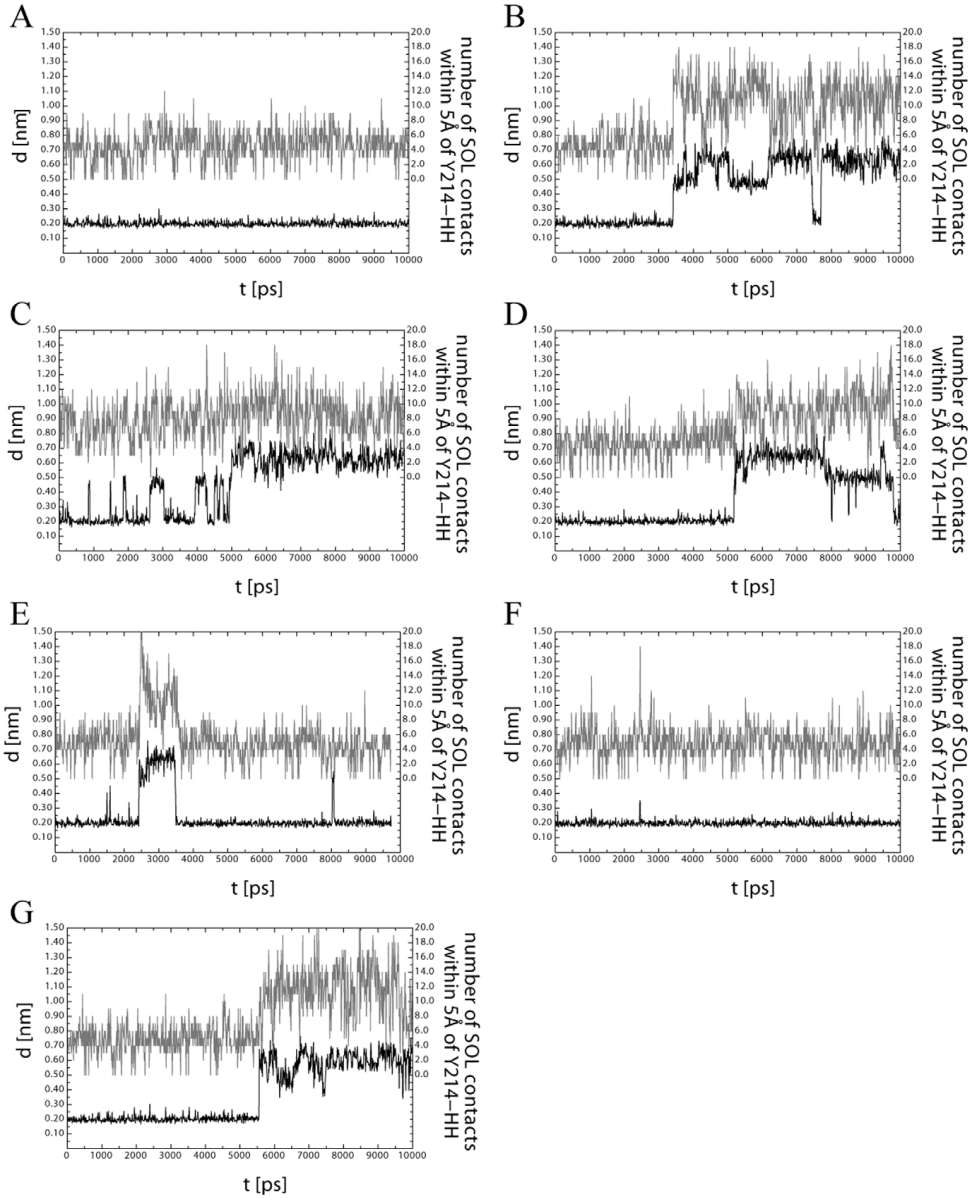
Results of the VDE structural integrity during 10 ns molecular dynamics simulations. Representative results of the molecular dynamics simulations of VDE in water show the hydrogen bond, i.e. the distance between ND1-H121 and HH-Y214 (black trace) and the amount of water within the central hydrophobic barrel of VDE (grey trace, solvent contacts within 0.5 nm of Y214-HH). Opening of the barrel structure is required for dimerization and activation of VDE. In (A) protonation states of VDE were chosen as calculated using continuum electrostatics, B–F) the five target residues, i.e. with a pK_a_ within the activation range of VDE have been fixed in their protonated state, one at the time: D98 (B), D117 (C), H168 (D), D206 (E), D86 (F). G) same analysis was performed with all four candidate residues for pH dependent conformational change while in their protonated state.

Figure 4 shows the distance between the atoms ND1 of H121 and HH of Y214 and the number of solvent contacts of Y214-HH during the 10 ns simulations. All simulations have been repeated independently three times from scratch (Figure S4). Figure 4A shows the results of VDE_pH7_ at pH 7 using the protonation states taken from Table S1, i.e. according to the pK_a_ calculations. The distance between H121 and Y214 is stable around 0.2 nm and the number Y214-HH solvent contacts remains around 5 in all three repetitions, indicating that the calculated pK_a_ values are sensible and that the central barrel motif is stable. As additional confirmation, the fluctuations of the alpha carbons during the simulations strongly correlate with the temperature factors observed in the molecular structure resolved by x-ray crystallography (Figure S5) supporting the pK_a_ calculations and the reliability of the setup in the molecular dynamic runs.

The same molecular dynamics simulations were also performed changing the protonation state of those five residues identified above as potential key players of VDE activation (Figure 4B-E). Four out of five candidate residues, if protonated, are able alone to induce a loosening of the hydrogen bond between H121 and Y214. The distance between these two atoms is found to increase in two steps from the original 0.2 to around 0.5 and 0.7 nm (Figure 4B and S4). It is worth underlining that in several cases during simulations the H121–Y214 bond was first broken and then re-formed suggesting its disruption is reversible. The protonation of these residues thus opens the possibility of a second protein conformation, which is in equilibrium with the first one.

Simulations also showed an increased solvent accessibility within the barrel during the time frame of the simulation (Figure 4, Figure S4). Interestingly, in all runs the increase in water molecules preceded the hydrogen bond rupture. This suggests a picture where the residue protonation induced some loosening of the barrel and an increased water penetration. These water molecules compete with the Tyrosin hydroxy group thus reducing the strength of the H121–Y214 hydrogen bond. The simulations also show that after breaking the hydrogen bond with H121, Y214 is hydrogen bonded to water.

Only in the case of protonation of D86, all independent molecular dynamic simulations showed neither destabilization of the central barrel nor any increase in water accessibility within the time frame of the simulation (Figure 4E, S4).

In order to shed more light on the importance of each residue in activation, the same simulations were performed changing the protonation state of all four residues at the same time. These analyses showed consistent results with respect to the disruption of the H121–Y214 hydrogen bond and with respect to the distance between ND1 and HH going from 0.2 to 0.5–0.7 nm. Shortly prior to this event, solvent is penetrating the hydrophobic barrel core as evidenced by the number of water molecules within 0.5 nm of Y212-HH, which increases from around 5 to more than 11 (Figure 4F). This is equal to an increase of 200–300 nm^2^ solvent accessible surface within the hydrophobic barrel and an increase of the volume from about 0.800 nm^3^ to up to 1.600 nm^3^.

### Conservation of Residues Putatively Involved in pH Activation in Different Organisms

**Figure 5:**
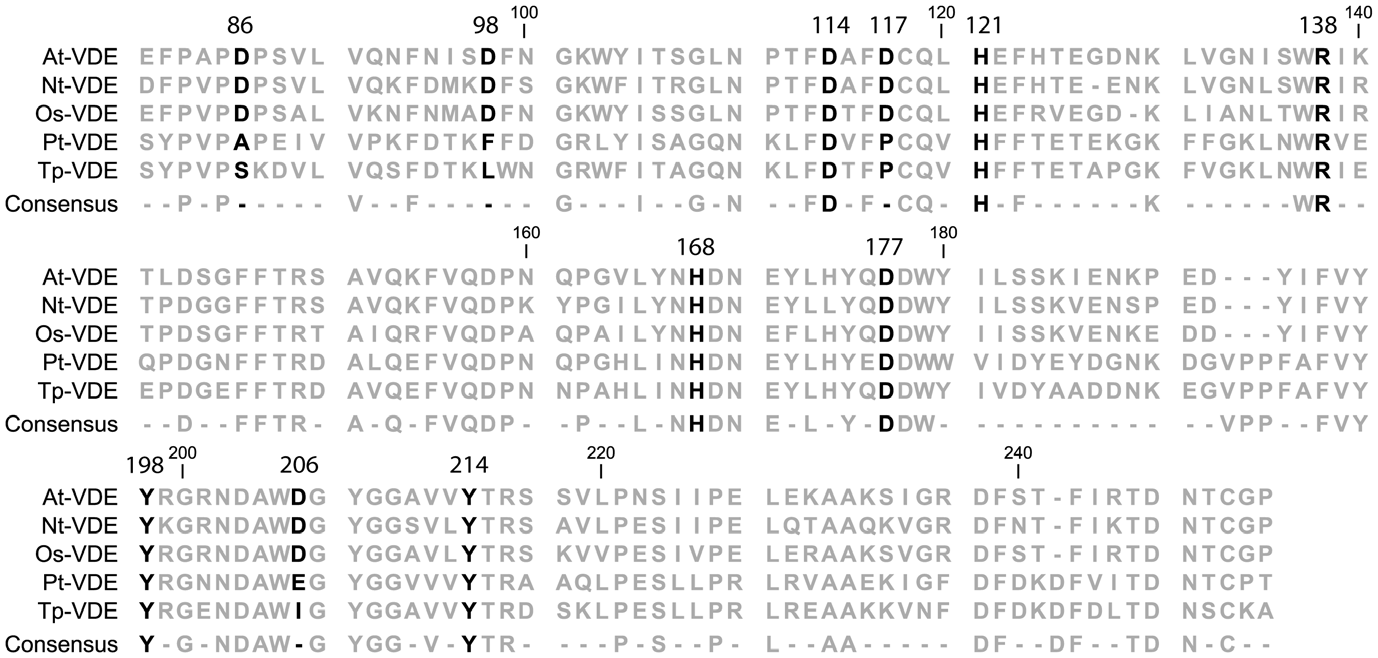
Sequence alignment of VDE from different plant and diatom species. ClustalW alignment of VDE sequences from plants (At, *Arabidopsis thaliana*, Nt, *Nicotiana tabacum*, Os, *Oryza sativa*) and diatoms (Pt, *Phaeodactylum tricornutum*, Tp, *Thalassiosira pseudonana*). Only the region corresponding to VDE_cd_, for which structural data is available is shown. Residues numbering refers to *Arabidopsis* mature protein. Residues discussed in the text are shown in black, while the others are in grey. Consensus sequence (100% identity) and conservation percentage are also shown.

VDE is found to be conserved in many different photosynthetic eukaryotes and among them are diatoms, which are particularly interesting because a) diatoms diverged from plants early during the evolution of photosynthetic eukaryotes [6], [15], [20] and b) the diatom VDE shows a major difference in its pH activation range. While the diatom VDE is already activated between a pH of 6.5 and 7, the plant isoform has no detectable activity above pH 6 [21], [22]. Their xanthophyll cycle activation has thus been differently optimized during evolution like as an adaptation to their natural environment. In order to assess if this optimization is reflected in the amino acid composition, VDE sequence of some vascular plants and diatoms is compared in Figure 5. All residues that are essential for the catalytic activity (D177 & Y198 [15]) or are important for the structural organization of VDE (D114, R138, H121 and Y214) are found to be conserved in plants and diatoms. The five putative residues involved in pH dependent activation are all found conserved in plants but not in diatoms. While H168 is conserved in all organisms, the four aspartates found in the plants VDE are replaced by non-ionizable amino acids in the diatoms form (Figure 5).

## Discussion

Violaxanthin De-Epoxidase (VDE) is a crucial enzyme for the photo-protective xanthophyll cycle as it is responsible of the synthesis of zeaxanthin, a carotenoid with a seminal role in the strong light response [1], [3]. VDE activation is triggered by the decrease in lumenal pH, which occurs when photosynthesis is saturated. The decrease in lumenal pH is the major signal for regulation of photosynthesis and its effect goes beyond the activation of xanthophyll cycle, e.g. being also fundamental for the activation of Non Photochemical Quenching, npq [7]. The molecular structure of the lipocalin domain resolved at different pH suggested that VDE undergoes a conformational change during activation, which is associated with the opening of the tight beta sheet barrel, dimerization and its binding to the membrane [14], [15]. In this study, the pH dependent activation of VDE has been investigated on a molecular level using a combination of *in silico* and site directed mutagenesis approaches.

Since VDE is activated by a decrease of lumenal pH, the residues involved in the molecular activation switch are expected to change their protonation state during pH transition, i.e. are expected have pK_a_s between 5.2 and 7. In order to identify target residues with these properties that could be used to elucidate the molecular activation switch, continuum electrostatics was employed to calculate the pK_a_ values from all residues of the inactive VDE. Five residues that fit the criteria for potential key players during activation were investigated in more detail. These residues were D86, D98, D117, D206 and H168.

To assess the pK_a_ values, residues putatively involved in pH activation were targeted by site directed mutagenesis. All residues tested but D86 showed a significant decrease in enzymatic activity, with H168A emerging as the variant with the highest impact, with only 20% residual activity left. This reduction in activity is not attributable to an alteration of the catalytic efficiency, since all residues are located far from the active site of the protein (Figure S6, [15]) and are not interacting with any of the residues with a key role in catalytic activity (Figure 2). With the exception of D117, they are also far from the monomer-monomer interface of VDE_pH5_ and not interacting directly or indirectly with residues involved in the dimer stabilization (Figure 1, 2B). A more likely explanation for the mutants phenotype, consistent with all data presented, is that these residues are involved in the pH dependent activation of VDE. In their absence, the activation process is less efficient, leading to a reduced amount of active VDE.

The analysis of VDE in different photosynthetic eukaryotes showed that the four residues involved in pH dependent activation are all conserved in plants but not in diatoms. While H168 is conserved in all organisms, the three aspartates that are found in the plants VDE are replaced by non-ionizable amino acids in the diatoms form (Figure 5). This difference is particularly interesting because VDE of diatoms is already activated between a pH of 6.5 and 7, while the plant isoform has no detectable activity above pH 6 [21], [22]. Since the aspartates found in plants have acidic pK_a_ values and thus are protonated only at lower pH, sequence analyses thus suggest a picture where in diatoms the H168 protonation, eventually accompanied by the protonation of other unidentified residues, is driving VDE activation already at higher pHs, while the activation of the plant VDE is shifted towards more acidic pHs due to the presence of the identified aspartates.

Molecular dynamics simulations were carried out to test the role of these residues with respect to the structural integrity of VDE. The amount of water entering the hydrophobic core barrel and the stability of the H121–Y214 hydrogen bond were monitored as indicators of the protein activation. From an electrostatic point of view, the disruption of the hydrogen bond and the protonation of H121 is an important prerequisite for the stability of the VDE dimer (Figure 2B). Molecular dynamic simulations of the VDE_pH7_ structure, protonated according to continuum electrostatic results showed a stable hydrophobic barrel during all independent simulations, showing little water entering the hydrophobic core structure and a stable hydrogen bond between H121–Y214. Similarly, the protonation of D86 did not yield any changes in the barrel structure or an increased amount of water within the barrel (Figure 4).

The structural integrity was however affected during molecular dynamics simulations with D98, D117, H168 or D206 locked in their protonated state, as expected upon change in pH to 5.2. More water molecules entering the beta barrel and a weakening of the H121–Y214 hydrogen bond (Figure 4) were found. Since the hydrogen bond destabilization was shown to be reversible and the destabilization occurred not equally frequently in all simulations, the destabilization can be seen as the first step during activation, which is in equilibrium with other steps that have not been taken into account. Evidently, the increased amount of water within the beta barrel will also change the pK_a_ values of several residues within the hydrophobic core. Such a change could currently not be included in the simulations. The disruption of the H121–Y214 hydrogen bond will certainly change the pK_a_ of H121 and its positive charge will most likely help to drive the necessary conformational changes required for dimerization.

A picture that emerges from the results presented here points to an activation mechanism where four out of the five potential activation residues are contributing to a conformational change as a result of a reduction in pH. This hypothesis is consistent with previous reports showing that VDE activation has a cooperative nature and involves between 4 and 5.3 protons [8], [21].

The four activation residues identified here are not necessarily the only residues of the pH dependent molecular activation switch of VDE. Firstly, pK_a_ calculations come with an intrinsic uncertainty [16] and secondly, the molecular structure that was used to determine the pK_a_ values was obtained by truncating the protein, i.e. the Glutamate-rich and Cysteine-rich domains of VDE were not considered. In fact, the quadruple mutant, where all four putative protonable residues have been substituted, still showed some small residual activity indicating the existence of at least one additional activation residue. Nevertheless, it has to be pointed out that structural data clearly show that VDE_cd_ alone is capable of undergoing the pH dependent conformational change and additional residues, if present, are part of the robust and redundant activation network described here.

**Figure 6:**
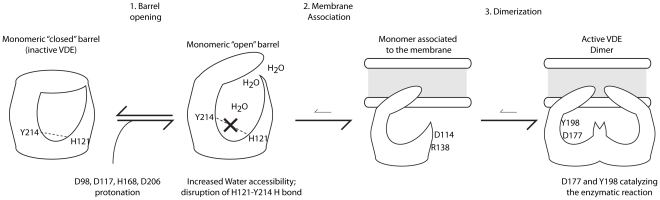
Model of VDE activation. A model of VDE activation according to presented data is shown. The first step is the transition from the closed barrel structure (experimental inactive VDE) to a more open structure, where the cavity is more accessible to water and where the H121–Y214 hydrogen bond is destabilized. In a second step the open monomer associates to the membrane and successively dimerizes (thanks to interactions involving D114 and R138), leading to the final active conformation (active VDE). Here Y198 and D177 are available to catalyze the enzymatic reaction. In the absence of D98, D117, H168 and D206, the formation of active dimer is less efficient but possible since steps 2 and 3 are dependent from other residues.

A model of the VDE activation integrating all results presented here and the results presented in the literature is shown in Figure 6. VDE is in its inactive form a soluble monomer that consists of a closed beta barrel structure as observable in the molecular structure of VDE_pH7_. Upon protonation of key residues the beta barrel structure is destabilized leading to a more open state, with water molecules entering the beta barrel, and to a destabilization of the H121–Y214 hydrogen bond, as suggested by molecular dynamics simulations. This “open” structure is then capable of interacting with the thylakoids membrane where its substrate violaxanthin is found. Since structural data on VDE lipocalin domain suggest that the protein is active as a dimer [14], this model can be integrated suggesting that VDE might dimerize at the membrane before yielding the fully active form.

The VDE transition between the closed and open form (step 1) is in equilibrium between the two conformations. The protonation of the four residues identified above shifts the equilibrium towards the “open” structure at low pH, thus increasing protein activation efficiency. On the contrary, when these residues are not protonated the closed conformation is more stable. This idea is consistent with molecular dynamics simulation where the protein structural destabilization was shown to be reversible (Figure 4).

It is important to underline that these four residues are neither implicated in the dimerization [14] nor in the catalysis of VDE [15]. Additionally, the electrostatic interaction node maps of the VDE_pH5_ structure show that out of the four identified activation residues only D117 is involved in the electrostatic driven stabilization of the dimeric structure. Thus, steps 2 and 3 of the activation, as hypothesized in Figure 6, will be possible even if the formation of the “open” VDE form is inefficient. The few enzyme molecules that are in the “open” form can still be fully catalytically active, as experimentally observed. This proposed model fits with the data presented here and explains why, even in the case of the quadruple mutant, VDE can catalyze violaxanthin de-epoxidation, i.e. can reach, although with an extremely reduced efficiency, its active conformation.

In conclusion, the investigation of the first activation steps of VDE presented here point to a complex yet robust activation network. The results indicate that a) each of the four identified key residues, D98, D117, D206 and H168 are already sufficient to induce the first step of the activation, i.e. opening of the central barrel structure prior to dimerization, and b) the four residues together produce a cooperative effect on the activation. In the light of these results, sequence comparison between plants and diatoms are in line with the fact that VDE is activated at higher pHs in diatoms compared to higher plants.

## Materials and Methods

The molecular structure of Violaxantin de-epoxidase lipocalin domain was resolved as a dimeric at pH 5 (3CQR.pdb, in the following called VDE_pH5_) and in the monomeric form at pH 7 (3CQN.pdb, VDE_pH7_). Structure files were taken from the protein data bank (http://www.pdb.org).

### Structure File Adjustments

Structural alignment between VDE_pH7_ and VDE_pH5_ was performed using p3d [23]. Residues 168–177 were copied from VDE_pH5_ into VDE_pH7_ since those were missing in the original structure.

### pK_a_ Calculations

Multi conformer continuum electrostatics (MCCE) version 2.4 [16] and DELPHI v.4 [17], [18] was used to calculate the pK_a_ values of residues in VDE_pH7_ structure using default settings. Three independent runs were performed. Averaged pK_a_ as well as the standard deviation for all ionizable residues can be found in Table S1.

### VDE Expression, Purification Site-directed Mutagenesis and Activity Tests

The construct expressing mature *A. thaliana* VDE was kindly provided by Prof. Yamamoto [12]. VDE expression and purification were done as in [15], as also described in Supplementary material. VDE sequence was mutated with QuickChange© Site directed Mutagenesis Kit, from Stratagene©. WT and mutants enzymatic activity was measured as described previously [15], [24] and the results were confirmed using HPLC [25]. Since substantial amount of contaminants are present in the VDE purification, protein amounts in all tests were verified to be equivalent for WT and mutants by Western blot with specific antibodies.

### Electrostatic Node Maps and Contact Maps

Electrostatic interaction node maps were created using networkX (http://networkx.lanl.gov) and pyGraphviz (http://networkx.lanl.gov/pygraphviz/), both calling Graphviz (http://www.graphviz.org/) subroutines. Python scripting language was used to translate the electrostatic interaction energies into vertices and edges. The interaction energies were calculated using mfe.py, a Python script that is part of the MCCE package. Interaction energies have been collected and averaged using a simple Python script. The width of the edge is scaled according to the strength of the interaction using penwidth = abs(InteractionEnergy) * (maxPenWidth – *minPenWidth)/(upperInteractionEnergyThreshold)*, with *maxPenWidth = 11px, minPenWidth = 0.2px* and *upperInteractionEnergyThreshold = 4 Kcal*. Green and red edges indicate stabilizing or de-stabilizing interactions, respectively. *K-core* analysis was performed using networkX.

### Molecular Dynamics

Molecular dynamic simulations were performed using Gromacs 4.0.3, double precision [26] and the amber force field 1994 [27]. All simulations were performed independently at least three times (see Supplementary Material). The volume of water inside the hydrophobic core structure of VDE_pH7_ was calculated using fpocket (http://pubs.acs.org/doi/abs/10.1021/jm100574m).

## Supporting Information

Figure S1 **Accessibility to VDE lipocalin cavity.** Top view of inactive VDE (A, red) and active VDE (B, blue) structures, showing the residues volume. Structures are shown with the same orientation. While the binding cavity is accessible in the case of active form (right), this is blocked in inactive protein (left), in particular by loop L1.
(TIF)

Figure S2 **Contact map of the VDE dimer.** Shown are the minimal distance in Å between residues of chain A and chain B from the VDE dimer.
(TIF)

Figure S3 **VDE WT and mutants purification and activity tests.** A) The construct expressing mature *Arabidopsis thaliana* VDE cloned in pQE60 was kindly provided by Prof. Yamamoto [12]. VDE WT and site-specific mutants were expressed in *E. coli* (Origami B strain, [28]). Cells with a 600-nm absorbance of 0.6 were induced with 1 mM IPTG (Isopropyl β-D-1-thiogalactopyranoside) for 5 hours at 37°C. Cells were thereafter centrifuged at 6000 g and 4°C for 10 min, resuspended in TRIS HCl pH 8, 250 mM NaCl, and lysed by sonication. VDE was then purified on a nickel affinity column (from Sigma©). Samples were run on 12% SDS-PAGE and then transferred to nitrocellulose membranes. VDE was detected with home-made antibody raised against *Arabidopsis thaliana* protein [29]. Protein loading for each mutant was modified to obtain a similar antibody signal as shown in the example reported. B) Mutants activity quantification by HPLC as in [25].
(TIF)

Figure S4 **Data from independent molecular dynamics simulations.** Simulations reported in figure 4 were repeated three times each. MCCE output files were formatted to the Gromacs/amber file format using p3d [23]. Initial structures were minimized in vacuum using first a conjugated then a steep gradient method. Relaxed structures were then solvated in a dodecahedron. 150 mM NaCl were added to neutralize the system and to achieve similar conditions to the MCCE runs. The box size was 225.83 nm^3^. The minimum distance between periodic images was at least 1.5 nm. The system included finally between 22384 and 22393 atoms. The system was then given time to “soak” in water for 70 ps, keeping the protein harmonically restraint and letting the water equilibrate around and into the protein. Finally, molecular simulations were performed using nose-hoover temperature coupling at 300 K and a coupling constant of 1 ps and Parrinello-Rahman pressure coupling of 1 bar, compressibility of 4.5e^−5^ bar^−1^ and a coupling constant of 1 ps. Electrostatic interactions were treated using the particle Mesh Ewald method [30] with an order of 8, a tolerance of 1e^−5^ and Fourier spacing of 0.1 nm. Initial velocity was created randomly following a Boltzmann distribution. All restrains were removed prior to 10 ns molecular dynamics simulation. The time step was 0.002 ps. Cut-off for VDW and electrostatic interaction were 1.5 nm and 1.0 nm, respectively. The volume of water inside the hydrophobic core structure of inactive VDE was calculated using fpocket (http://pubs.acs.org/doi/abs/10.1021/jm100574m).
(TIF)

Figure S5 **rmsf vs structure temperature factor.** Top: Averaged rmsf of the alpha carbons during molecular simulations of inactive VDE. Error bars show standard deviation of the three independent runs. pKas of the residues are taken from Table S1 and inactive VDE was protonated accordingly to pH7. Bottom: temperature factor of the alpha carbons in the inactive VDE expressed as deviation from mean in units of overall std.
(TIF)

Figure S6 **Position of ionizable residues with respect to VDE active site.** A) Position of ionizable residues with respect to enzyme active site in the pH 5 structure (active VDE) is shown, focusing on one monomer. Ionizable residues are shown in grey sticks, residues fundamental for enzymatic activity (D177 and Y198) in green, violaxanthin in orange and ascorbate in yellow. B) Same structure as in A after a 90° rotation.
(TIF)

Table S1 pKa values calculated for inactive VDE monomer at pH 7 (3CQN.pdb). Three independent runs were performed and here average and standard deviation values are reported. Between brackets is shown the sign of the eventual charged state. Residues showing pKa between 5 and 7 (± 0.5) are highlighted. Residues numbers are taken from deposited structure and correspond to the *Arabidopsis* mature protein.
(XLS)

Table S2 pKa values calculated for dimeric and active form of VDE (3CQR.pdb). Three independent runs were performed and here average and standard deviation valued are reported. Between brackets is shown the sign of the eventual charged state and the letter identifies the polypeptide chain. Residues showing pKa between 5 and 7 (± 0.5) are highlighted. Residues numbers are taken from deposited structure and correspond to the *Arabidopsis* mature protein.
(XLS)

Table S3 A k-cores decomposition (http://arxiv.org/abs/cs.DS/0310049) can be exploited to identify the most relevant nodes in the map. Here data obtained using a 0.2 Kcal mol^−1^ threshold is reported. Graph density is 0.058599695586 and 0.0291378074994 for active and inactive VDE, respectively. Residues with K values below 3 and 5 were not reported for inactive and active VDE. The first letter identifies the polypeptide chain.
(XLS)
