# Supplementary figures and images for: Identification of Key Residues for pH Dependent Activation of Violaxanthin De-Epoxidase from Arabidopsis thaliana
Source: PLoS One. 2012 Apr 27;7(4):e35669. doi: 10.1371/journal.pone.0035669 (PMC3338714; doi:10.1371/journal.pone.0035669)

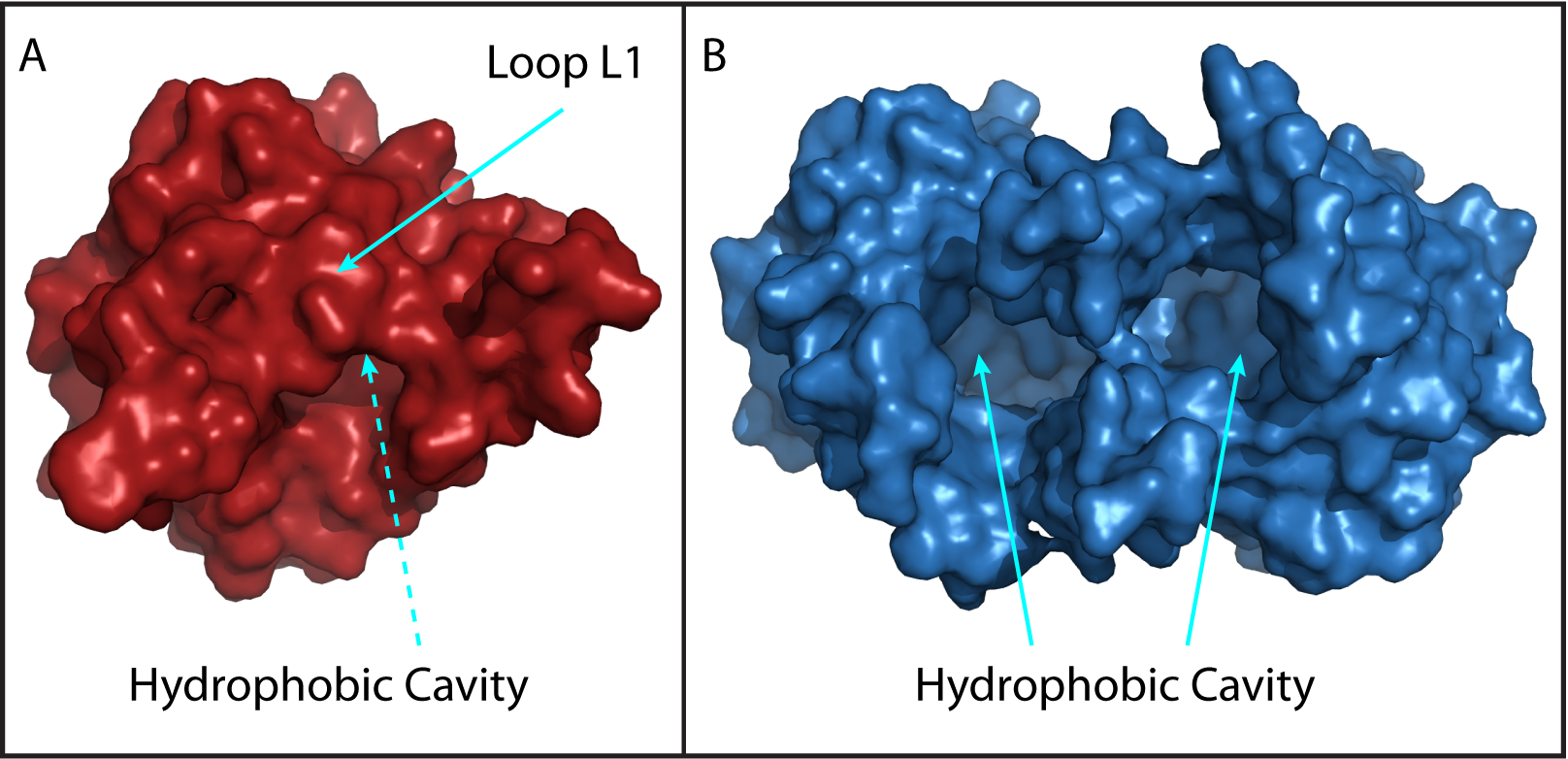

Supplement: Figure S1 — Accessibility to VDE lipocalin cavity. Top view of inactive VDE (A, red) and active VDE (B, blue) structures, showing the residues volume. Structures are shown with the same orientation. While the binding cavity is accessible in the case of active form (right), this is blocked in inactive protein (left), in particular by loop L1. (TIF) [file pone.0035669.s001.tif]

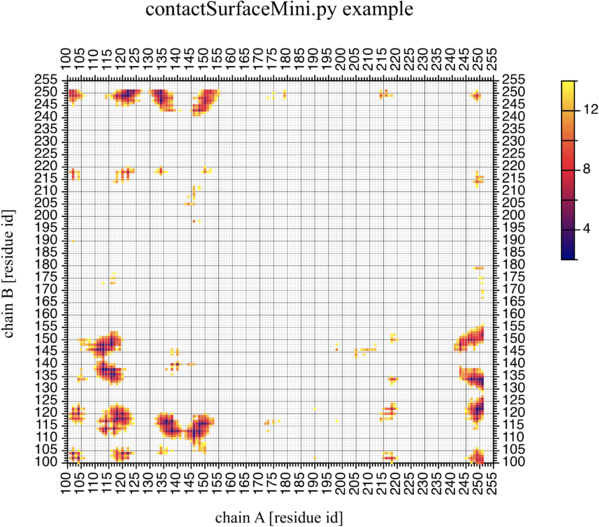

Supplement: Figure S2 — Contact map of the VDE dimer. Shown are the minimal distance in Å between residues of chain A and chain B from the VDE dimer. (TIF) [file pone.0035669.s002.tif]

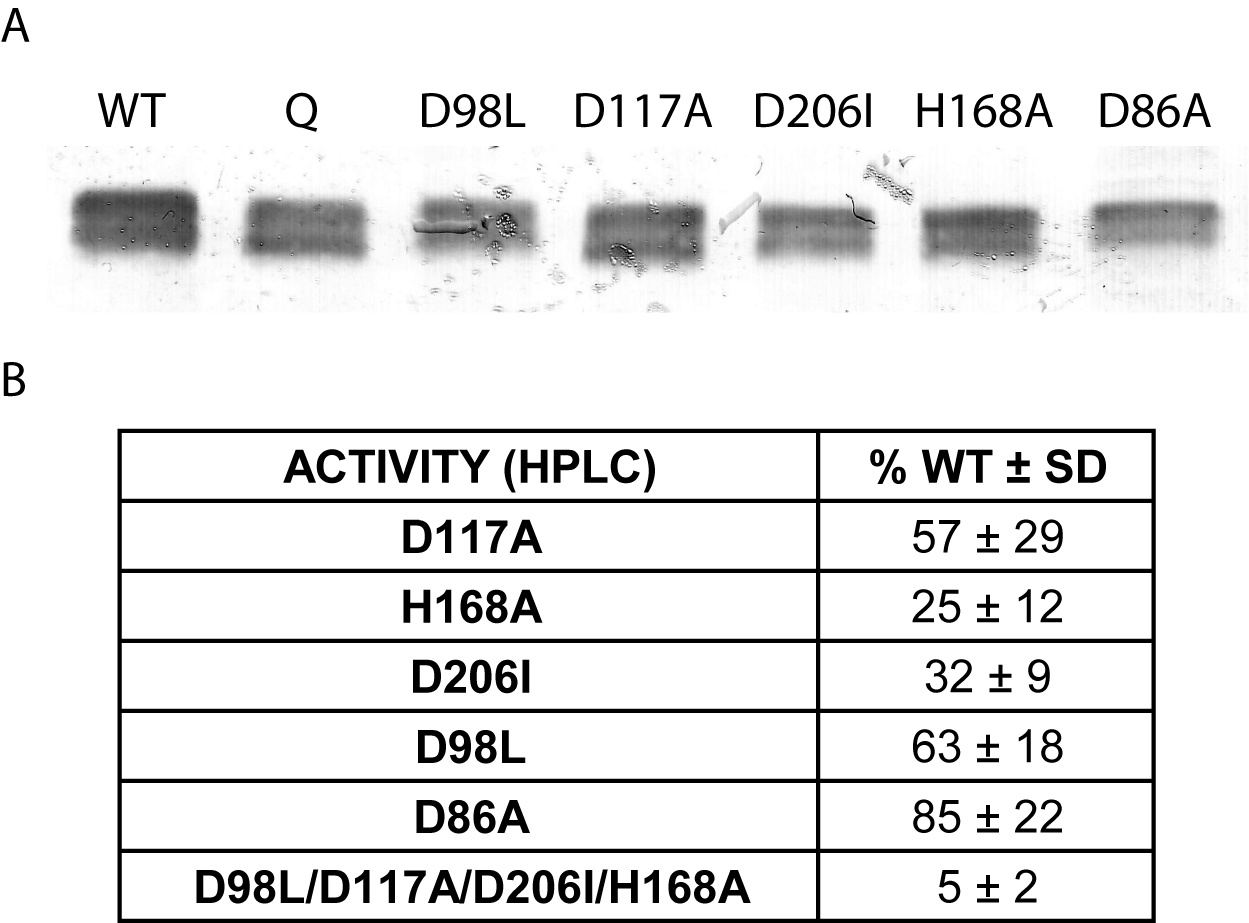

Supplement: Figure S3 — VDE WT and mutants purification and activity tests. A) The construct expressing mature Arabidopsis thaliana VDE cloned in pQE60 was kindly provided by Prof. Yamamoto [12]. VDE WT and site-specific mutants were expressed in E. coli (Origami B strain, [28]). Cells with a 600-nm absorbance of 0.6 were induced with 1 mM IPTG (Isopropyl β-D-1-thiogalactopyranoside) for 5 hours at 37°C. Cells were thereafter centrifuged at 6000 g and 4°C for 10 min, resuspended in TRIS HCl pH 8, 250 mM NaCl, and lysed by sonication. VDE was then purified on a nickel affinity column (from Sigma©). Samples were run on 12% SDS-PAGE and then transferred to nitrocellulose membranes. VDE was detected with home-made antibody raised against Arabidopsis thaliana protein [29]. Protein loading for each mutant was modified to obtain a similar antibody signal as shown in the example reported. B) Mutants activity quantification by HPLC as in [25]. (TIF) [file pone.0035669.s003.tif]

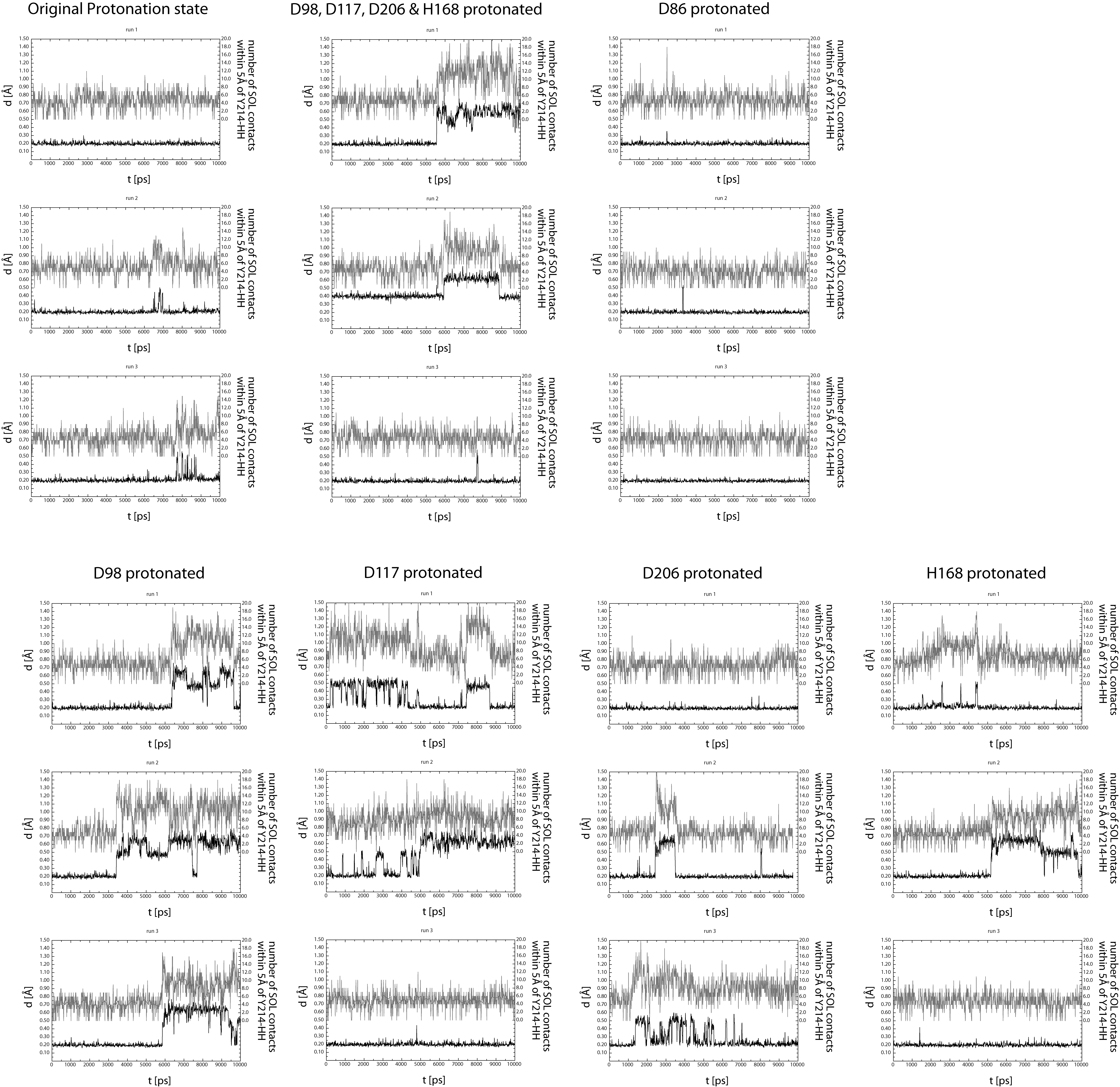

Supplement: Figure S4 — Data from independent molecular dynamics simulations. Simulations reported in figure 4 were repeated three times each. MCCE output files were formatted to the Gromacs/amber file format using p3d [23]. Initial structures were minimized in vacuum using first a conjugated then a steep gradient method. Relaxed structures were then solvated in a dodecahedron. 150 mM NaCl were added to neutralize the system and to achieve similar conditions to the MCCE runs. The box size was 225.83 nm3. The minimum distance between periodic images was at least 1.5 nm. The system included finally between 22384 and 22393 atoms. The system was then given time to “soak” in water for 70 ps, keeping the protein harmonically restraint and letting the water equilibrate around and into the protein. Finally, molecular simulations were performed using nose-hoover temperature coupling at 300 K and a coupling constant of 1 ps and Parrinello-Rahman pressure coupling of 1 bar, compressibility of 4.5e−5 bar−1 and a coupling constant of 1 ps. Electrostatic interactions were treated using the particle Mesh Ewald method [30] with an order of 8, a tolerance of 1e−5 and Fourier spacing of 0.1 nm. Initial velocity was created randomly following a Boltzmann distribution. All restrains were removed prior to 10 ns molecular dynamics simulation. The time step was 0.002 ps. Cut-off for VDW and electrostatic interaction were 1.5 nm and 1.0 nm, respectively. The volume of water inside the hydrophobic core structure of inactive VDE was calculated using fpocket (http://pubs.acs.org/doi/abs/10.1021/jm100574m). (TIF) [file pone.0035669.s004.tif]

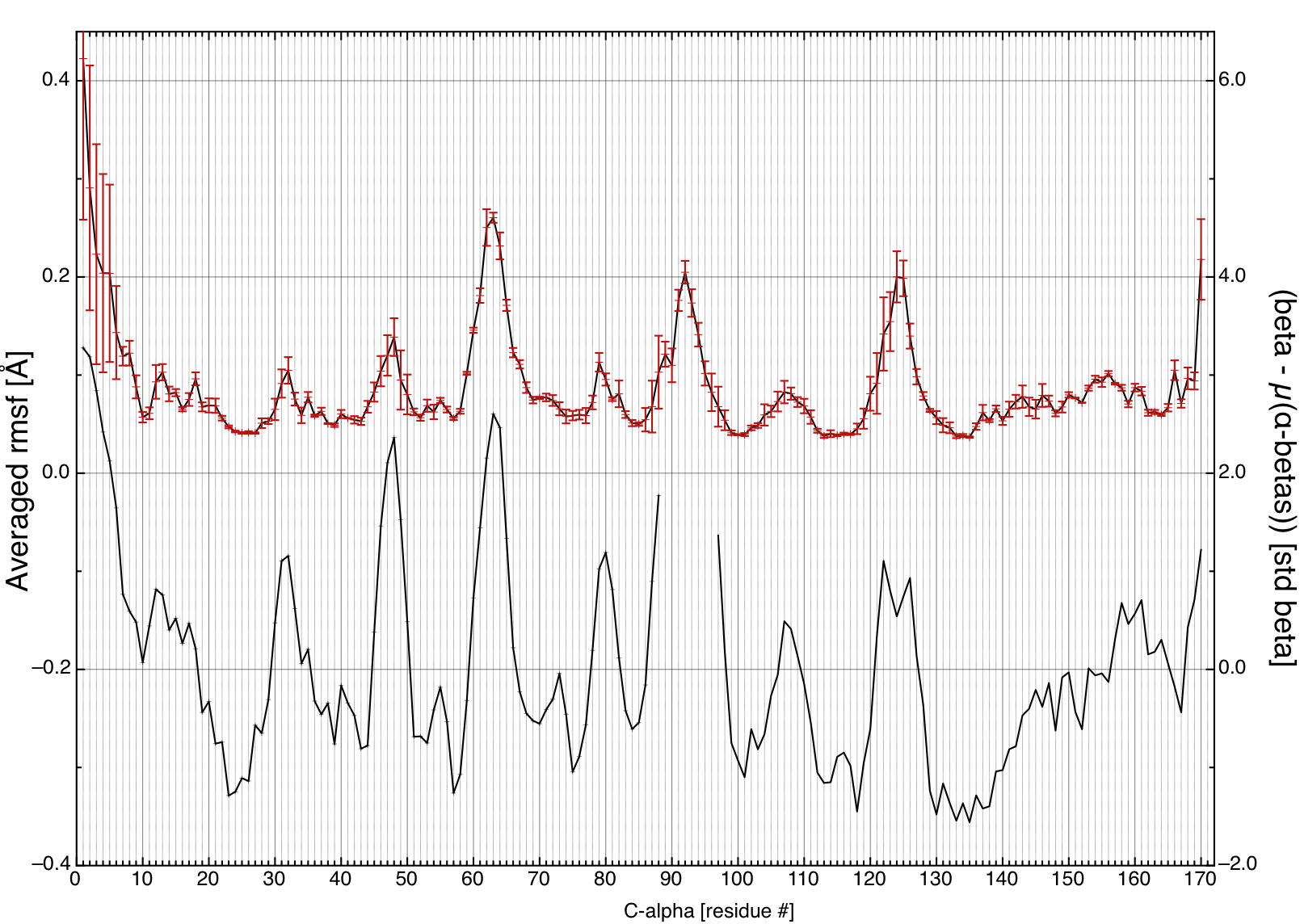

Supplement: Figure S5 — rmsf vs structure temperature factor. Top: Averaged rmsf of the alpha carbons during molecular simulations of inactive VDE. Error bars show standard deviation of the three independent runs. pKas of the residues are taken from Table S1 and inactive VDE was protonated accordingly to pH7. Bottom: temperature factor of the alpha carbons in the inactive VDE expressed as deviation from mean in units of overall std. (TIF) [file pone.0035669.s005.tif]

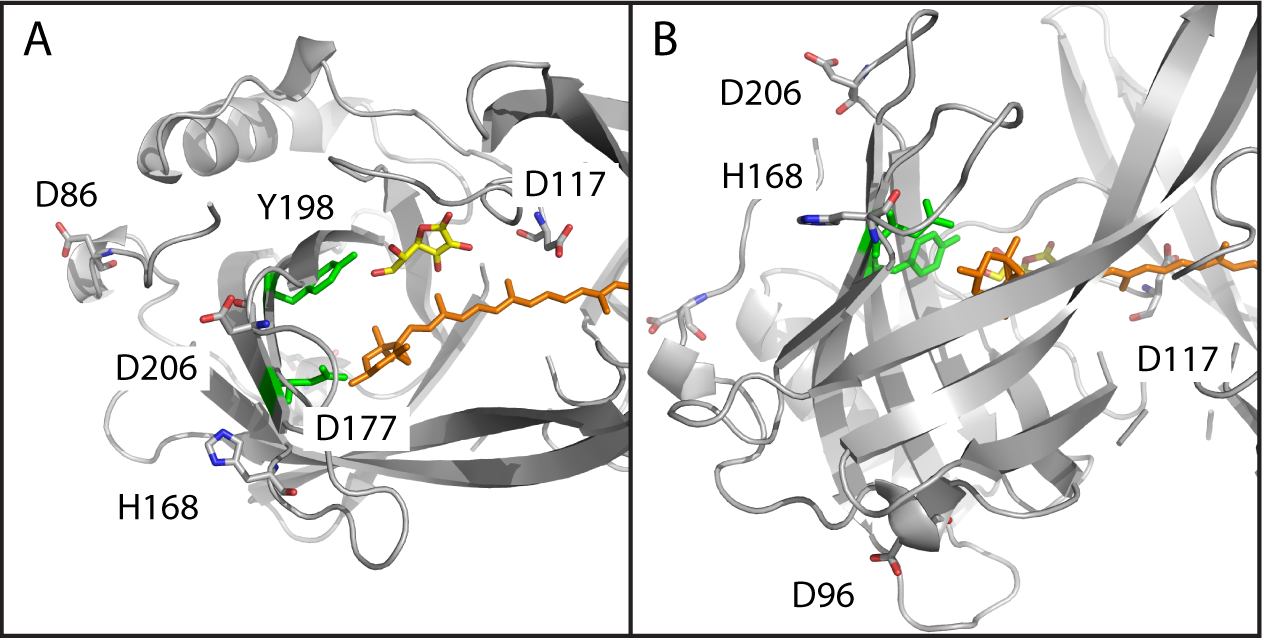

Supplement: Figure S6 — Position of ionizable residues with respect to VDE active site. A) Position of ionizable residues with respect to enzyme active site in the pH 5 structure (active VDE) is shown, focusing on one monomer. Ionizable residues are shown in grey sticks, residues fundamental for enzymatic activity (D177 and Y198) in green, violaxanthin in orange and ascorbate in yellow. B) Same structure as in A after a 90° rotation. (TIF) [file pone.0035669.s006.tif]
